# Brain functional connectivity correlates of autism diagnosis and familial liability in 24-month-olds

**DOI:** 10.1186/s11689-025-09621-9

**Published:** 2025-07-18

**Authors:** John R Pruett Jr., Alexandre A Todorov, Zoë W Hawks, Muhamed Talovic, Tomoyuki Nishino, Steven E Petersen, Savannah Davis, Lyn Stahl, Kelly N Botteron, John N Constantino, Stephen R Dager, Jed T Elison, Annette M Estes, Alan C Evans, Guido Gerig, Jessica B Girault, Heather Hazlett, Leigh MacIntyre, Natasha Marrus, Robert C McKinstry, Juhi Pandey, Robert T Schultz, William D Shannon, Mark D Shen, Abraham Z Snyder, Martin Styner, Jason J Wolff, Lonnie Zwaigenbaum, Joseph Piven, for the IBIS Network, J. Piven, J.R. Pruett, R.T. Schultz, J. Pandey, J. Parish-Morris, B. Tunc, W. Guthrie, J.T. Elison, J.J. Wolff, C.A. Burrows, H.C. Hazlett, M.D. Shen, J.B. Girault, R. Grzadzinski, S.R. Dager, A.M. Estes, T. St. John, D.W.W. Shaw, K.N. Botteron, R.C. McKinstry, J.N. Constantino, N. Marrus, A. Rocca, C. Chappell, L. Zwaigenbaum, M.R. Swanson, M.A. Styner, G. Gerig, A.C. Evans, L.C. MacIntyre, S. Torres-Gomez, S. Das, K. Truong, H. Volk, M.D. Fallin, S.S. Jeste, K.E. MacDuffie

**Affiliations:** 1 Department of Psychiatry, Washington University School of Medicine, St. Louis, MO 63110 USA; 2 Brooklyn Health, 195 Montague St 14, Brooklyn, NY 11201 USA; 3 Department of Neurology, Washington University School of Medicine, St. Louis, MO 63110 USA; 4 Division of Biology and Biomedical Sciences, Washington University School of Medicine, St. Louis, MO 63110 USA; 5 Mallinckrodt Institute of Radiology, Washington University School of Medicine, St Louis, MO USA; 6 Department of Psychiatry and Behavioral Sciences, Emory University School of Medicine, Atlanta, GA 30322 USA; 7 Department of Radiology and Bioengineering, University of Washington, Seattle, WA 98195 USA; 8 Institute of Child Development, University of Minnesota, Minneapolis, MN 55455 USA; 9 Speech and Hearing Sciences, University of Washington, Seattle, WA 98105 USA; 10 McConnell Brain Imaging Center, Montreal Neurological Institute, Montreal, QC Canada; 11 Department of Computer Science and Engineering, Tandon School of Engineering, New York University, Brooklyn, NY 11201 USA; 12 Department of Psychiatry, University of North Carolina at Chapel Hill, Chapel Hill, NC 27599 USA; 13 McGill Centre for Integrative Neuroscience, Montreal Neurological Institute- Hospital, McGill University, Montreal, QC Canada; 14 Mallinckrodt Institute of Radiology, Washington University School of Medicine, St. Louis, MO 63110 USA; 15 Center for Autism Research, The Children’s Hospital of Philadelphia, University of Pennsylvania, Philadelphia, PA 19104 USA; 16 Department of Psychiatry, University of Pennsylvania, Philadelphia, PA 19104 USA; 17 Co-Founder, BioRankings, St. Louis, MO 63119 USA; 18 Professor Emeritus of Biostatistics in Medicine, Washington University School of Medicine, St. Louis, MO 63110 USA; 19 Department of Educational Psychology, University of Minnesota, Minneapolis, MN 55455 USA; 20 Department of Pediatrics, University of Alberta, Edmonton, AB T6G 2R3 Canada; 21 The Carolina Institute for Developmental Disabilities, University of North Carolina at Chapel Hill, Chapel Hill, NC 27599 USA

**Keywords:** Infant, Functional connectivity, MRI, Default mode network, Familial

## Abstract

**Background:**

fcMRI correlates of autism spectrum disorder (ASD) diagnosis and familial liability were studied in 24-month-olds at high (older affected sibling) and low familial likelihood for ASD.

**Methods:**

fcMRI comparisons of high-familial-likelihood (HL) ASD-positive (HLP, *N* = 23) and ASD-negative (HLN, *N* = 91), and low-likelihood ASD-negative (LLN, *N* = 27) 24-month-olds from the Infant Brain Imaging Study (IBIS) Network were conducted, employing object oriented data analysis (OODA), support vector machine (SVM) classification, and network-level fcMRI enrichment analyses.

**Results:**

OODA (alpha = 0.0167, 3 comparisons) revealed differences in HLP and LLN fcMRI matrices (*p* = 0.012), but none for HLP versus HLN (*p* = 0.047) nor HLN versus LLN (*p* = 0.225). SVM distinguished HLP from HLN (accuracy = 99%, PPV = 96%, NPV = 100%), based on connectivity involving many networks. SVM accurately classified (non-training) LLN subjects with 100% accuracy. Enrichment analyses identified a cross-group fcMRI difference in the posterior cingulate default mode network 1 (pcDMN1)– temporal default mode network (tDMN) pair (*p* = 0.0070). Functional connectivity for implicated connections in these networks was consistently lower in HLP and HLN than in LLN (*p* = 0.0461 and 0.0004). HLP did not differ from HLN (*p* = 0.2254). Secondary testing showed HL children with low ASD behaviors still differed from LLN (*p* = 0.0036).

**Conclusions:**

24-month-old high-familial-likelihood infants show reduced intra-DMN connectivity, a potential neural finding related to familial liability, while widely distributed functional connections correlate with ASD diagnosis.

**Supplementary Information:**

The online version contains supplementary material available at 10.1186/s11689-025-09621-9.

## Introduction

Recent reviews [1–3] report resting-state functional connectivity magnetic resonance imaging (fcMRI) differences, often involving the default mode network (DMN), in children and adults with autism spectrum disorder (ASD) versus those with typical development. However, these also highlight inconsistencies in findings, in part due to small sample sizes, heterogeneity, age range, and methods for fcMRI data acquisition and processing (see also: [4–6]).

We know very little about DMN functional connectivity differences in infants and toddlers. In this study we focus on a specific early age, the 24-month window. Brain functional architecture changes drastically over the first years of life [7–13], and infants, even those who will eventually be diagnosed with autism at later ages, typically do not manifest the range of behaviors that meet diagnostic criteria for ASD [14]. These behaviors emerge and consolidate during the second and third years of life. There are a growing number of fcMRI studies in infants and toddlers with ASD or at high likelihood for developing ASD (e.g [15–25]). These papers variously report cortical and thalamocortical connectivity patterns and network-level fcMRI correlates of emerging ASD-relevant behaviors in the first years of life. DMN components are frequently implicated in this body of work (see Figure S5 in [21]). Understanding early neural correlates of ASD may inform early identification [26, 27], open up the possibility of presymptomatic intervention trials (e.g [28]; for review: [29]), establish biomarkers for demonstrating treatment engagement [30, 31]; and, provide potential insights regarding brain mechanisms.

The prevalence of autism is estimated to be one in 36 [32]. The prospective study of infants at high familial likelihood (HL) for later developing autism, by virtue of having and older sibling with autism [33], provides an opportunity to examine potential neural correlates of ASD as it emerges in the first years of life, prior to the consolidation of symptoms into the full syndrome, as defined in DSM-5. Approximately one in 5 children at high familial likelihood [34] will develop ASD, making the study of HL infants a more cost-effective strategy for exploring early presymptomatic neural markers than the study of a population infant sample. Autistic traits are heritable and continuously distributed in the general population [35]. There is a range of outcomes that are subthreshold for full ASD diagnosis and genetically related to ASD, which occur at rates increased over the population, in siblings who do not themselves develop ASD. These outcomes include phenotypes with milder but qualitatively similar features to those which define ASD (e.g., cognitive, motor and language delays [36–38] and other psychiatric disorders e.g., anxiety, depression, and attention-deficit/hyperactivity disorder [39–41]).

The objectives of the present study are to evaluate whether functional connectivity at brain-wide and/or network levels distinguishes cross-group ASD diagnostic and familial likelihood status at 24 months. Three groups are considered: high likelihood ASD positive (HLP), for children who meet diagnostic criteria for ASD and are siblings of older autistic individuals; high likelihood ASD negative (HLN); and low likelihood ASD negative (LLN). We previously reported fcMRI correlations with ASD-relevant dimensionally measured behaviors in overlapping samples: initiation of joint attention [15], walking and gross motor development [16], and restricted and repetitive behavior [17]. However, our prior sample had low power for the categorical diagnostic outcome-based (HLP vs. HLN vs. LLN) comparisons reported here. Advances in fcMRI processing procedures over the last five years (used for [21]) made the present analyses possible. We followed a pre-specified analytic plan that included a set of brain-wide and network-level statistical tests.

## Methods and materials

### Participants

We analyzed fcMRI and behavioral data from toddlers who participated in The Infant Brain Imaging Study (IBIS) at four sites: the University of North Carolina, Children’s Hospital of Philadelphia, Washington University School of Medicine in St Louis, and the University of Washington. All families provided written informed consent. LORIS (Longitudinal Online Research and Imaging System) [42] served as the hub for data management.

Subjects contributed fcMRI and diagnostic outcome data at 24 months. High-likelihood (HL) toddlers had at least one older sibling diagnosed with ASD. Low-likelihood (LL) toddlers had one or more older sibling(s) with typical development and no first- or second-degree relatives with ASD or intellectual disability. Experienced clinicians applied a DSM-IV-TR checklist to all available testing (including the Autism Diagnostic Observation Schedule) and interview data to generate clinical best-estimate diagnoses of ASD [37], yielding three groups: high-likelihood-positive (HLP) with a clinical best estimate ASD diagnosis at 24 months, high-likelihood-negative (HLN), and low-likelihood-negative (LLN) toddlers. There were no low-likelihood-positive toddlers with useable 24-month fcMRI data. A measure of ASD severity was generated from Autism Diagnostic Observation Schedule (ADOS and ADOS-2) modules by computing calibrated severity scores (CSSs: [43]) across all symptoms.

### MRI acquisition

fcMRI data were acquired with cross-site calibrated 3 T Siemens MAGNETOM TIM Trio scanners with 12 channel head coils. A sagittal three-dimensional T2 weighted sequence (TE = 497 ms, TR = 3200 ms, 1 × 1 × 1 mm voxels) enabled registration with blood oxygen level dependent (BOLD) images. An identical protocol was used at each site, namely a gradient echo planar image acquisition (TE = 27 ms, TR = 2500 ms, voxel size 4 × 4 × 4 mm voxels). Subjects contributing data for these analyses had at least two 6.25 min BOLD runs acquired during natural sleep [15].

### fcMRI processing

fcMRI processing has been described previously [9], as have the updates [21, 23] that enabled increased yield for clean, high-quality processed data, allowing for the present previously unreported 24-month diagnostic outcome group-based fcMRI comparisons. Briefly, raw data were preprocessed using an inhouse suite of imaging software (https://4dfp.readthedocs.io/en/latest/). BOLD data were corrected for within-frame inter-slice temporal differences and systematic odd/even frame banding. Multiple BOLD runs were registered within-run and across-runs, then registered to an age-specific target atlas using an affine transformation. Due to the contrast characteristics of infant images, the target registration of infant BOLD images leverages only the T2 volume, whereas our older subject pipelines use both T1 and T2. EPI distortions were corrected using a calculated Field Map. To minimize interpolation of data, each frame was transformed into atlas space using a single matrix transformation (BOLD ➔ T2 ➔ age specific target ➔ atlas target). The images were then normalized to have an intensity mode of 1000.

These standardized fMRI images were then post-processed using strategies outlined in [44] that correspond to type “36P + scrub” in Ciric et al. 2017 [45]. Frame-wise displacement (FD) values were calculated from the inter-frame registration above at a radius of 50 mm, and were used to censor the data at FD < 0.2 mm [44]. The censored data were then voxel-wise demeaned and detrended. Nuisance waveforms were regressed out using cross-frame movement metrics (rotation, translation, and expansion terms [46]) and time series of non-interest (whole brain, white matter, and cerebrospinal fluid, and their first derivatives). Censored frames were replaced with interpolated values based on spectral analysis [44, 47] prior to temporal bandpass filtering at 0.009 Hz < f < 0.08 Hz. Finally, a Gaussian filter (6 mm FWHM) was applied. All censored/interpolated frames were removed prior to the generation of the correlation values. Recent processing improvements include optimization of atlas registration, use of an average volume generated from all movement censored frames to improve registration, calculated field map distortion correction ([48, 49]; cf. https://4dfp.readthedocs.io/en/latest/), and correction of a bandpass padding error that minimally affected prior processing. These data are available for public release through the NDA if readers are interested in trying their own processing strategies.

### Regions of interest (ROI) and fcMRI matrix construction

Time series were generated for 230 functionally defined regions of interest (ROIs) as described in Pruett et al. [9] and Table S4 of Hawks et al. [21]. Please see [9] for a detailed description of ROI specification. Briefly, these 230 infant ROIs are a subset of the “Power 264 ROIs” [50], augmented with non-overlapping ROIs from the Philip et al., 2012 autism fMRI meta-analysis [51], that fit properly on infant anatomy at studied ages. Subjects included in analyses provided a minimum of 150 uncensored BOLD frames. Functional connectivity was estimated by Fisher z-transformed Pearson correlations between pairs of clean ROI time series. Historical, preliminary analyses demonstrated the sensitivity of machine learning classification to variation in the number of frames per subject used in the construction of fcMRI matrices. We thus required all subjects to contribute exactly 150 low motion frames (6.25 min: see [9] for precedent) for the SVM analyses reported in this paper (machine learning for fcMRI enrichment secondary vetting used the all-good-frame data: 6.25 or more minutes per subject). The 150 frames for primary SVM analyses were selected randomly across a nine-minute time period of concatenated BOLD runs for each subject.

### Functional networks

A standard community detection approach was used to group brain ROIs into networks. Specifically, a 24-month functional brain network solution was generated with Infomap [52], as implemented in MATLAB release 2015a (The MathWorks, Inc.). Two ROIs (TAL [17, −79, −34)], MNI [16.85, −79.89, −34.36]; and TAL [34, −67, −33], MNI [34.72, −67.08, −34.45]) could not be assigned to a network and were excluded from the enrichment analyses (but used in other analyses). The remaining 228 ROIs were assigned to 17 networks of size 3 (SAL) to 36 (DAN) ROIs. Structure-specific thresholding was applied to give a better chance for subcortical and cerebellar ROIs to integrate into brain-wide networks [53, 54]. We generated a consensus network structure across graph edge densities [54] and assigned network names by comparing our solution to other existing solutions (e.g [15, 21]).

### General analytic procedures

As stated, analyses were performed according to a pre-specified analytic plan.

Tests on jackknifed correlation matrices and those with object oriented data analysis (OODA) were conducted in R [55, 56]; machine learning used the Python *sklearn* package [57]; and fcMRI enrichment analyses were performed in Python using locally developed software. We did not correct for multiple comparisons across categories of analysis, but we do so, where appropriate, within specific analyses (e.g., OODA).

### Brain-wide statistical tests

#### Correlation jackknife

This analysis tested whether an individual’s matrix was more similar to those from their own group than to those from the other groups. We used a statistical jackknife procedure [61] to calculate correlations between (i) each participant’s vectorized correlation matrix and (ii) the vectorized mean correlation matrices for each of the three groups. Group-mean correlation matrices used data from n = 23 participants (smallest group size) to avoid potential biases due to unequal group size. A participant’s data were not included in the mean-correlation for his or her own group. Outliers three standard deviations from the sample mean were removed (N = 1 participant). Repeated measures analysis of variance (rmANOVA) examined main effects of and interactions between individual participant matrix (HLPi, HLNi, LLNi) and group mean matrix (HLPg, HLNg, LLNg) on correlation estimates. Empirical p-values were computed by randomly permuting the participants’ group assignments. We hypothesized that individuals would be more similar to their own group than to the other groups.

#### Group-level network structure

The Infomap community detection procedure was used to generate group-level network solutions [52]. We used the normalized mutual information (NMI) index, a measure of shared information, to quantify differences in network structure between groups [58]. This analysis tests for group-level differences in network architecture, which result from a non-linear transform on the correlations and may lead to different interpretations of potential group differences than tests on the correlations themselves (correlation jackknife, above, and object oriented data analysis, below).

#### Individual subject network structure

This analysis tests whether *subject-specific* network structures are more similar for subjects in the same group than for subjects in different groups. Infomap was used to generate a network solution for each subject and one for each group and jackknifed group (i.e., with an individual removed from his/her own group solution). Solutions were generated at two edge-densities (0.04, 0.08) so that the analyses would be performed on graphs with the same number of edges. We calculated the normalized mutual information (NMI) between each individual’s network structure and (i) the individual’s own within-group jackknifed network structure, and (ii) the other group’s network structure. Non-parametric tests were used to test for within- and between-group NMI differences, with p-values determined using permutation testing.

#### Object oriented data analysis (OODA)

Object Oriented Data Analysis [59, 60] was used to test for group differences in connectivity at the matrix (rather than ROI pair) level. OODA provides a whole-brain hypothesis testing approach for group-level fcMRI differences. OODA complements support vector machine-based classification involving entire matrices. Omnibus-level OODA results can be decomposed to see which network-level effects are driving whole brain results. The distance between matrices was assessed using Euclidean distance. Multidimensional scaling (MDS) plots were generated to visualize clustering and potential separation of groups of matrices. The significance threshold was set at 0.0167 (= 0.05/3– i.e., Bonferroni correction) in pairwise group comparisons. Follow-up analyses assessed which networks significantly contributed to the omnibus results.

#### Support vector machine

This analysis tested whether brain-wide fcMRI data can be used to classify groups, and– importantly– whether a fixed version of a trained classifier can perform accurately with data not used in training. Support vector machine (SVM) was used for two-group classification (e.g., HLP vs. HLN), using nested leave-one-out cross-validation (followed by out-of-sample testing– i.e., with subjects not used at all in training), a linear kernel with balanced feature weighting, and t-test feature filtering based on nominal (*p* < 0.05) significance. Confidence intervals for accuracy, positive predictive value (PPV), and negative predictive value (NPV) were obtained via bootstrapping. We identified ROI-pairs as important for accurate SVM classification according to the number of leave-one-out folds in which they were retained. This analysis was repeated 1,000 times on permuted data to assess the statistical significance of the classifier’s performance metrics. SVM provides an analytic complement to the other approaches that we used, and it enables examination of connections important for accurate cross-group classification, as well as their distribution within and across networks. Most importantly, after running SVM using a leave-out cross-validation, we crystallized the resulting machines into one fixed classifier that we then used to test *completely set-aside data that were never used in training*. We believe this sort of out-of-sample generalization test is a critical step toward (our future goal of) developing generalizable machine learning classifiers for autism outcome using MRI data acquired during the presymptomatic period in the first year of life.

### Network-level statistical tests

#### fcMRI enrichment

We used fcMRI enrichment analysis [21, 23] to test the hypothesis that particular network-network pairs would present with a higher proportion of ROI-pairs where mean connectivity differed across the three groups (HLP, HLN, LLN). We computed ANOVA F tests for each of the 26,335 ROI pairs, with the top 5% identified as “hits.” Permutation of group labels was used to calculate empirical p-values. Prior simulation work indicates randomization *p* < 0.001 = a strong enrichment signal– approximating brain-wide alpha = 0.05; and randomization *p* < 0.01 = interesting (see [21, 23]). To validate promising network-pair enrichment signals, we used the functional connections for all ROI-pairs in interesting-to-strong network-pairs to predict a related variable, ADOS CSS (using linear regression with leave-one-out-cross-validation). This secondary validation (again, please see [21]) procedure provides a “sanity check” on enrichment screening results, but it is *not* meant to be a stand-alone machine learning analysis. We accepted the enrichment result if the expected mean squared error (MSE) for this machine learning, secondary vetting procedure was significantly lower (*p* = 0.05) than in randomly shuffled data. We then conducted enrichment on post hoc Tukey-corrected t-tests for network pairs with a significant three-group F test that also survived this secondary validation test to identify potential pairwise group differences (e.g., HLP vs. HLN).

#### ROI membership in individual networks

This analysis tested for cross-group differences in ROI membership within each network. Here, a maximally inclusive (across all subjects) set of ROIs was generated for each network (e.g., ROIs populating the default mode network for any subject, across all groups). We then evaluated the cross-group statistical significance (randomization analysis: 10,000 shuffles) of the Jaccard index (which quantifies shared versus distinct members of different sets) for each network’s ROI membership using randomization.

## Results

Of 203 subjects with BOLD data, 141 (69%) passed all phases of quality control and were included in the final sample. 84 subjects from this sample overlap with subjects in Eggebrecht et al. (*n* = 107) [15], Marrus et al. (*n* = 99) [16], and McKinnon et al. (*n* = 107) [17] at the 24-month timepoint for clean fcMRI data. 23 HLP, 91 HLN, and 27 LLN subjects with complete fcMRI and behavioral data (see Table 1) were analyzed in this study. Sex ratios were consistent with longstanding reports of male > female sex ratio in ASD: HLP (78% male), HLN (57% male), LLN (59% male) [32]. Some analyses (fcMRI enrichment testing for familial likelihood differences) are limited to subjects with ADOS CSS = 1 (54 HLN, 1 HLP, and 21 LLN). SVM classification used subjects with 150 frame matrices: 22 HLP, 87 HLN, 26 LLN.

**Table 1:** Participant characteristics

	All Frame Data		ML 150-Frame Data	
	N	%	N	%
Sex				
Male	86	60.99	81	60
Female	55	39.01	54	40
Outcome group				
Low-likelihood negative	27	19.15	26	19.26
Male	16	59.26	15	57.69
Female	11	40.74	11	42.31
High-likelihood negative	91	64.54	87	64.44
Male	52	57.14	49	56.32
Female	39	42.86	38	43.68
High-likelihood positive	23	16.31	22	16.30
Male	18	78.26	17	77.27
Female	5	21.74	5	22.73
	Mean	SD	Mean	SD
Age at time of scan (in months)	24.39	0.76	24.39	0.77
Number of BOLD frames (after scrubbing)	266.87	65.87	150	0
24-month ADOS Severity Score CSS	2.02	1.73	2.03	1.74
Low-likelihood negative	1.23	0.51	1.24	0.52
High-likelihood negative	1.45	0.81	1.46	0.81
High-likelihood positive	5.05	1.94	5.10	1.97

Characteristics are presented for participants with 24-month functional connectivity magnetic resonance imaging (fcMRI) data. All Frame Data: matrices created from all useable BOLD frames after motion cleaning; ML 150-Frame Data: matrices created from exactly 150 frames per subject to avoid bias in primary machine learning (ML) analyses (please see methods)

### Brain-wide analyses

rmANOVAs failed to identify significant cross-group differences in jackknife correlations with vectorized matrices.

Pairwise cross-group NMI index differences for group network structure (HLN vs. LLN = 0.80; HLP vs. HLN = 0.77; HLP vs. LLN = 0.78) were not different from those expected by chance.

Following our pre-specified analytic plan, a jackknife test on NMI for individual subject-level Infomap solutions was evaluated for potential group differences in brain-wide network structure. Consistent with the findings described above for examinations of NMI for Infomap results run on group mean matrices, no significant results were observed.

**Fig. 1:**
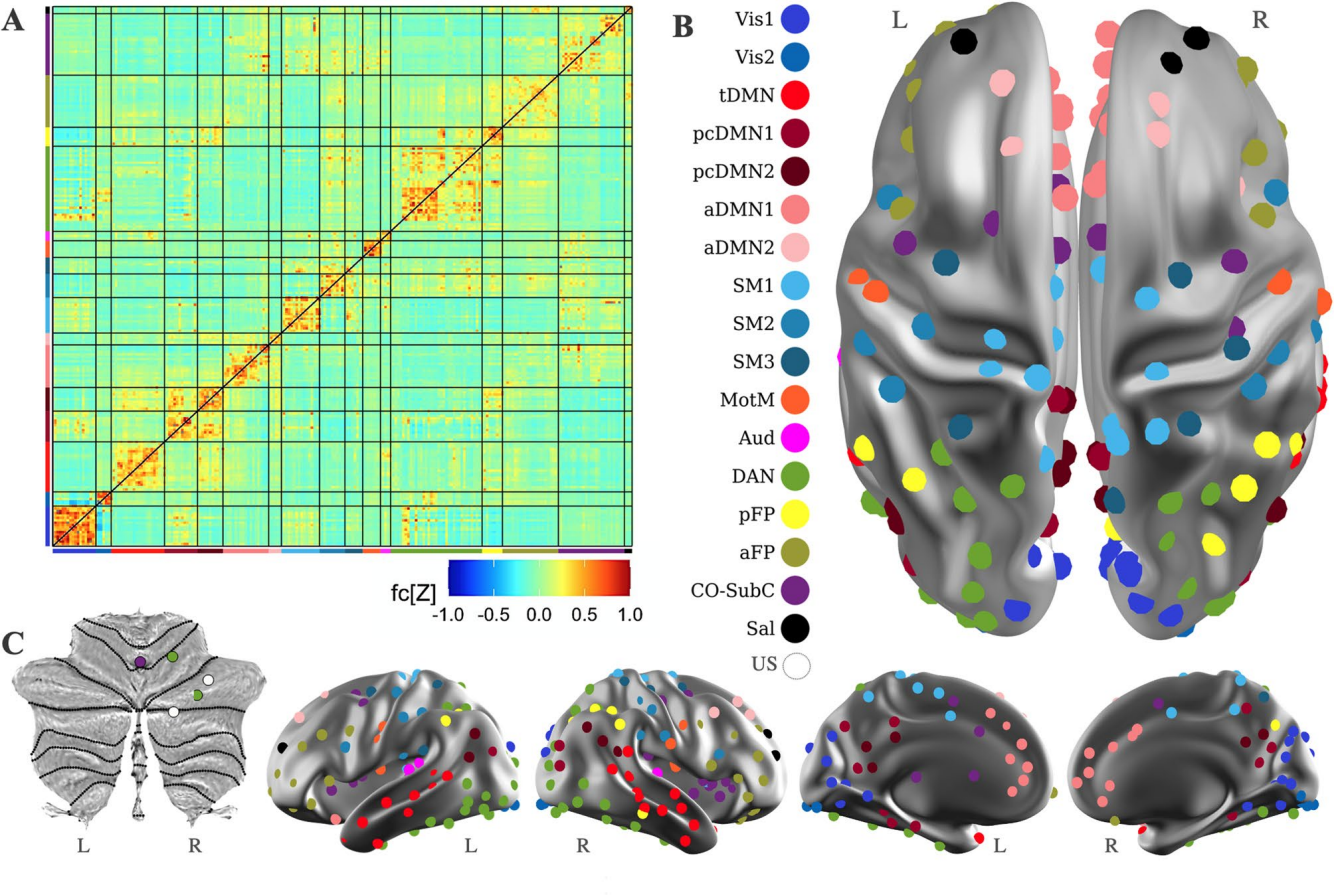
Functional network architecture in 24-month-old infants. **A **Average correlations for 230 spherical regions of interest (ROIs). The ROIs are sorted by network. **B **ROIs brain map (dorsal, lateral, and medial views). Colors correspond to network assignments. **C **Cerebellar ROIs on a flattened cerebellar surface [62]. aDMN1: anterior default mode network 1; aDMN2: anterior default mode network 2; aFP: anterior frontoparietal network; Aud: auditory network; CO-SubC: cingulo-opercular-subcortical network; DAN: dorsal attention network; MotM: motor-mouth network; pcDMN1: posterior cingulate default mode network 1; pcDMN2: posterior cingulate default mode network 2; pFP: posterior frontoparietal network; Sal: salience network; SM1: somatomotor network 1; SM2: somatomotor network 2; SM3: somatomotor network 3; tDMN: temporal default mode network; US: unspecified (not assigned); Vis1 and Vis2: visual 1 and 2

For use in subsequent analyses, since no reliable group-level network structure differences were identified, a single all-group network structure was created using all available clean fcMRI data from LLN, HLN, and HLP subjects (Fig. 1). This all-group network structure was used to contextualize the object oriented data analysis (OODA) and the fcMRI enrichment tests.

**Fig. 2:**
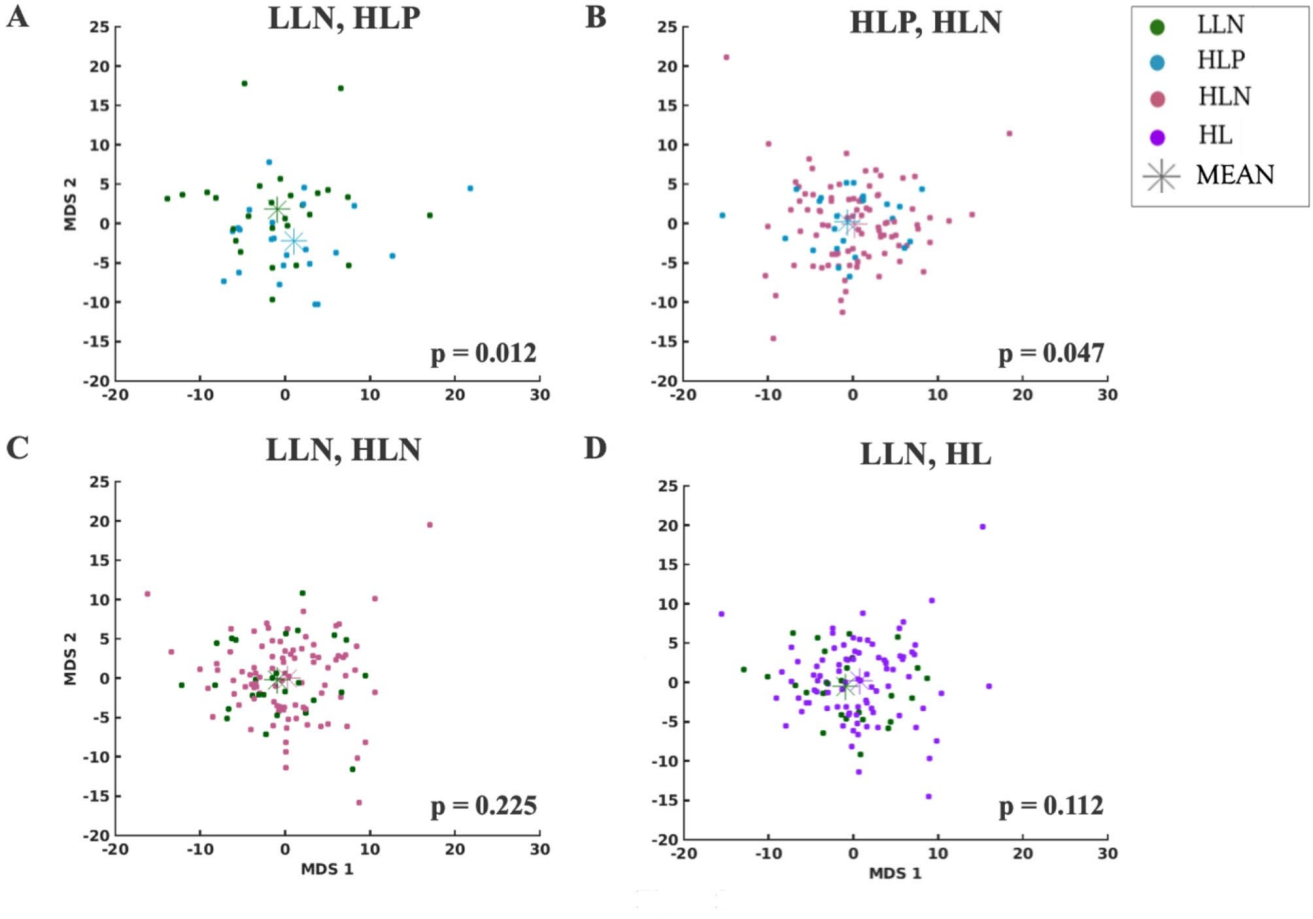
Multi-dimensional scaling (Euclidean distance) indicates (**A**) some separation (*p* = 0.012) between fcMRI correlation matrix cluster means (colored asterisks) for high-likelihood positive (HLP) and low-likelihood negative (LLN); but (**B**) not for HLP versus high-likelihood negative subjects (HLN; *p* = 0.047); nor for (**C**) HLN versus LLN subjects (*p* = 0.225). **D** Overall, the HL and LL groups did not differ significantly from one another (*p* = 0.112). Group label colors in figure inset

OODA analyses of brain-wide fcMRI group differences revealed significant differences between HLP and LLN fcMRI matrices (*p* = 0.012). HLP and HLN matrices and HLN and LLN matrices did not differ (*p* = 0.047 and 0.225, respectively). Multidimensional scaling plots (Fig. 2) show separation between the HLP and LLN groups, but not between HLP and HLN, nor between HLN and LLN. Follow-up OODA analyses could not identify particular networks driving the significant omnibus HLP-LLN result.

**Fig. 3:**
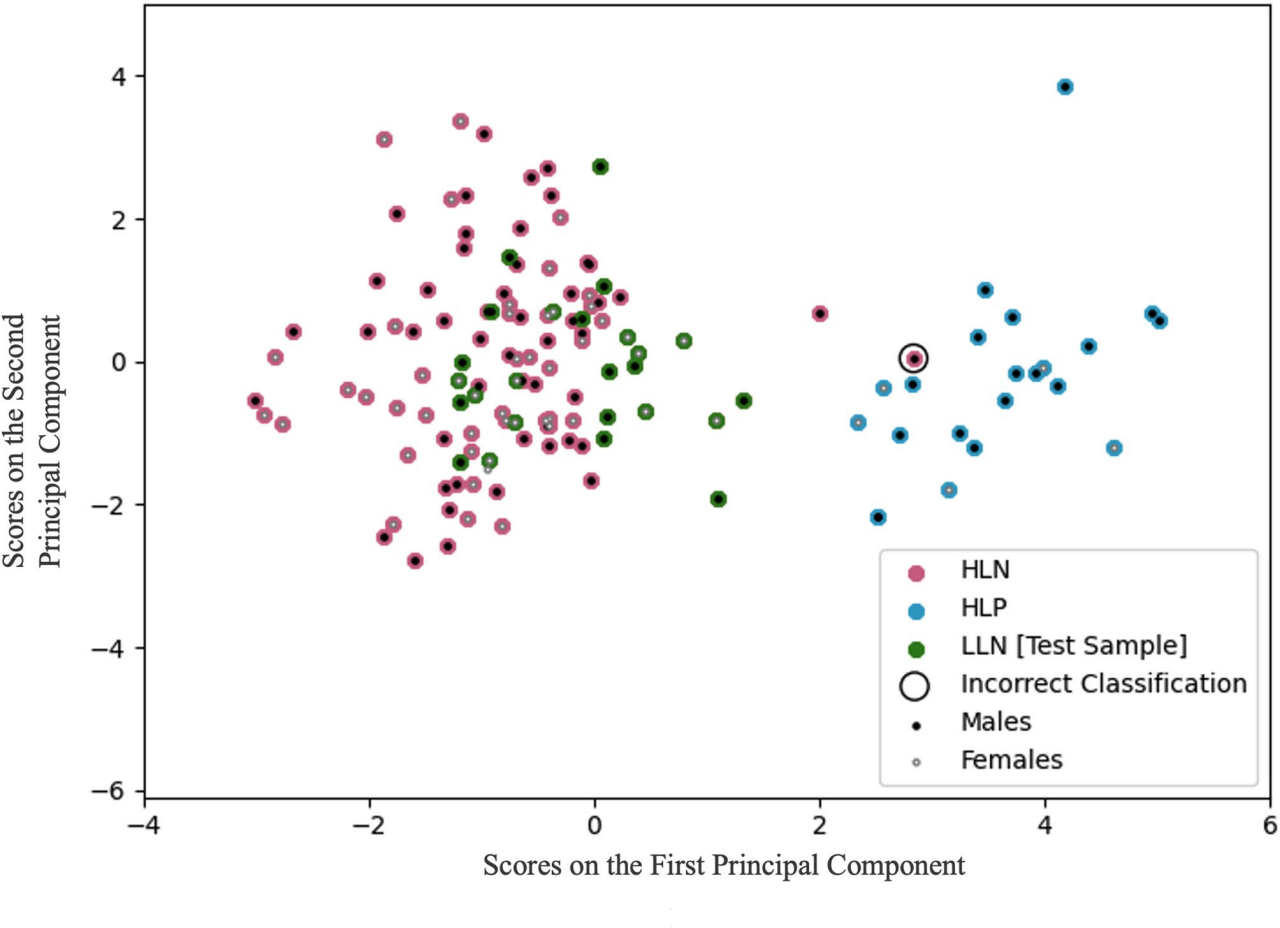
Scores on the first two principal components, computed on the set of features retained by the crystallized support vector machine classifier. The classifier, trained on high-likelihood positive and high-likelihood negative subjects only, was highly accurate (overall = 99.1%, males = 98.5%, females = 100%). LLN subjects were excluded from the training and were all correctly classified as unaffected. There is clear separation of HLP and HLN/LLN subjects, with only one incorrect positive classification (circled). Negative predictive values: overall = 100%, male = 100%, female = 100%. Positive predictive values: overall = 95.6%, male = 94.4%, female = 100%

The support vector machine (SVM) classifier clearly distinguished between HLP and HLN subjects (Fig. 3), with accuracy = 99.1% (95% CI: 97.2 − 100%); PPV = 95.6% (95% CI: 91.7–100%); and NPV = 100% (96.7–100%). The observed PPV and NPV were significantly higher than in randomized data (*p* < 0.001; 1000 permutations; Figure S1). LLN matrices were not used in building the SVM. The crystallized (i.e., training weights fixed) version of the classifier correctly labeled all of these LLN subjects as “ASD-negative” (Fig. 3).

**Fig. 4:**
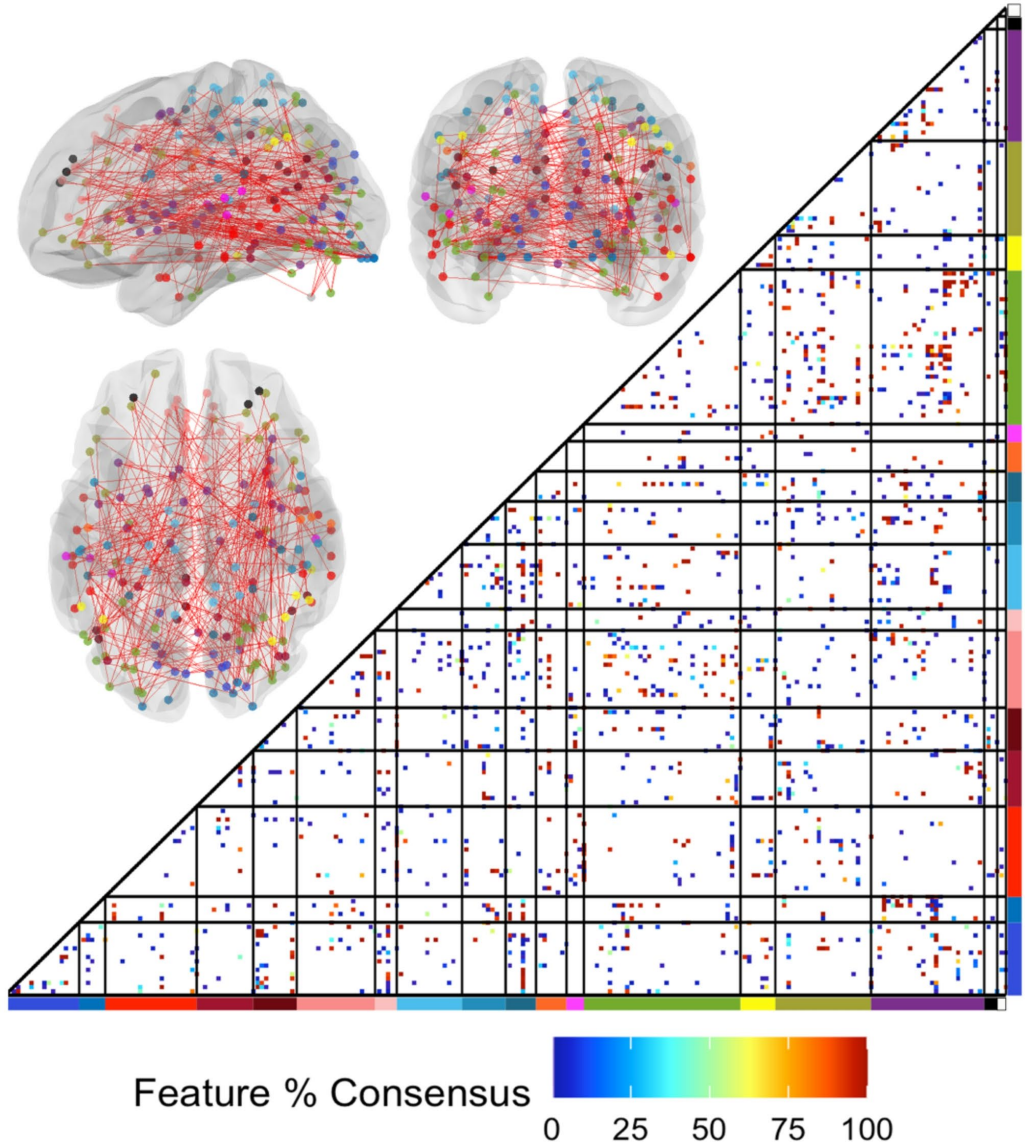
Connections important for accurate support vector machine (SVM) classification. The brain-wide support vector classifier was trained with high-likelihood positive and high-likelihood negative subjects. Consensus features (across leave-out folds) important for accurate classification are depicted on an adjacency matrix, and the 100% consensus features are shown on lateral, posterior, and dorsal views of the brain. Colors indicate network membership (see Fig. 1)

SVM performance was accurate for both males and females: only one male was misclassified during the training of the classifier, no males or females incorrectly classified within the testing dataset. The SVM consensus features were widely scattered across the brain (Fig. 4).

### Network-level analyses

**Fig. 5:**
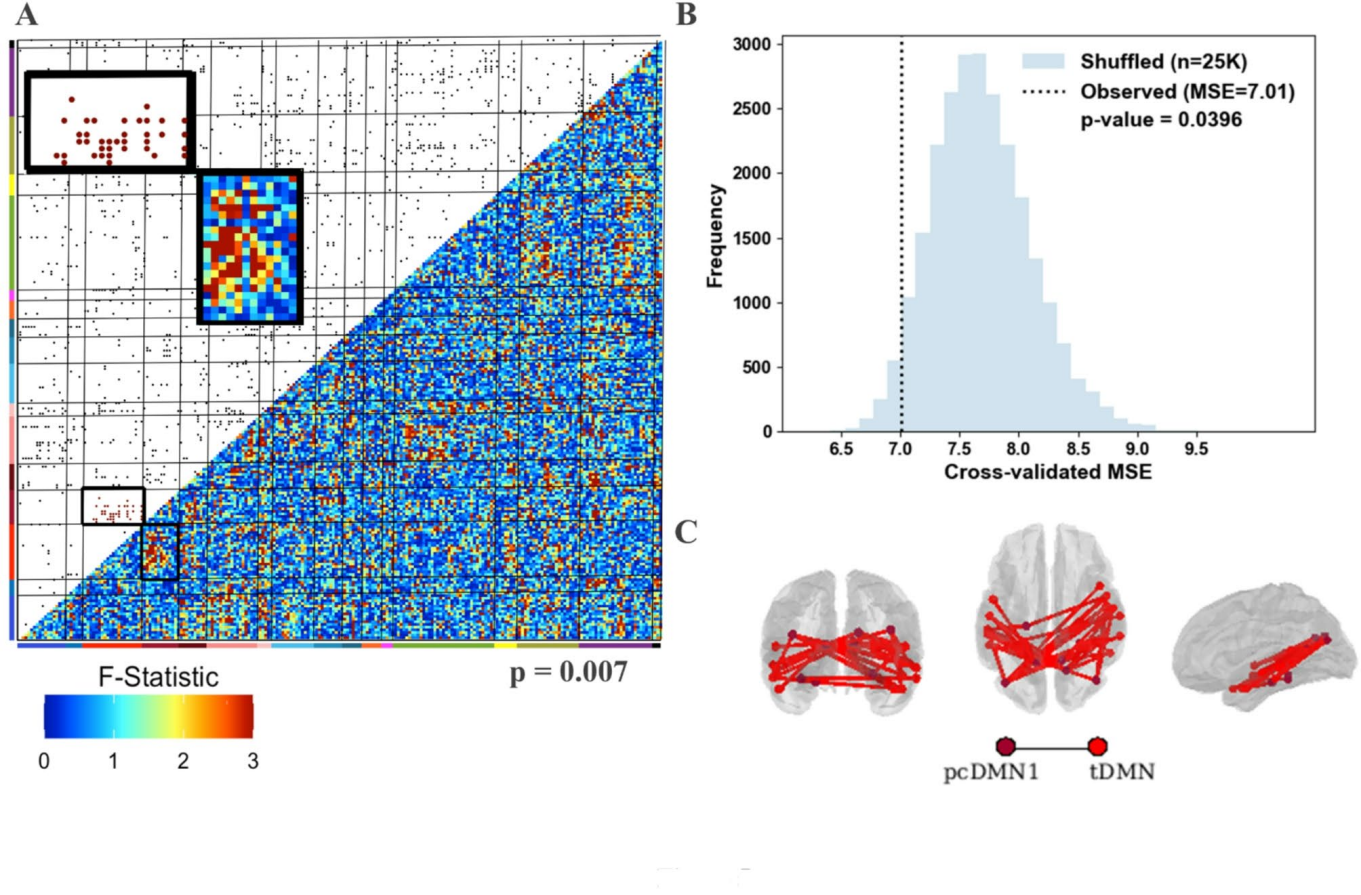
Enrichment analyses and secondary validation. A 3-group (HLP, HLN, LLN) ANOVA screen for functional connection differences suggests a clustering of nominally significant group differences for ROI pairs in the posterior cingulate default mode network 1 (pcDMN1)– temporal default mode network (tDMN) network pair (*p* = 0.007). **A **Lower triangle: ANOVA F-statistics. Upper triangle: 5% threshold applied to the F-statistics. The 230 ROIs are sorted by network (defined in Fig. 1). Black border: pcDMN1- tDMN. **B **Functional connections in the pcDMN1-tDMN network pair were used to predict ADOS CSS (five-fold cross-validated linear regression) with a Mean Squared Error of 7.01 (empirical *p* = 0.0396, on 25,000 permutations). **C **Locations of implicated connections within pcDMN1 - tDMN visualized on posterior, dorsal, and lateral views of the brain

**Fig. 6:**
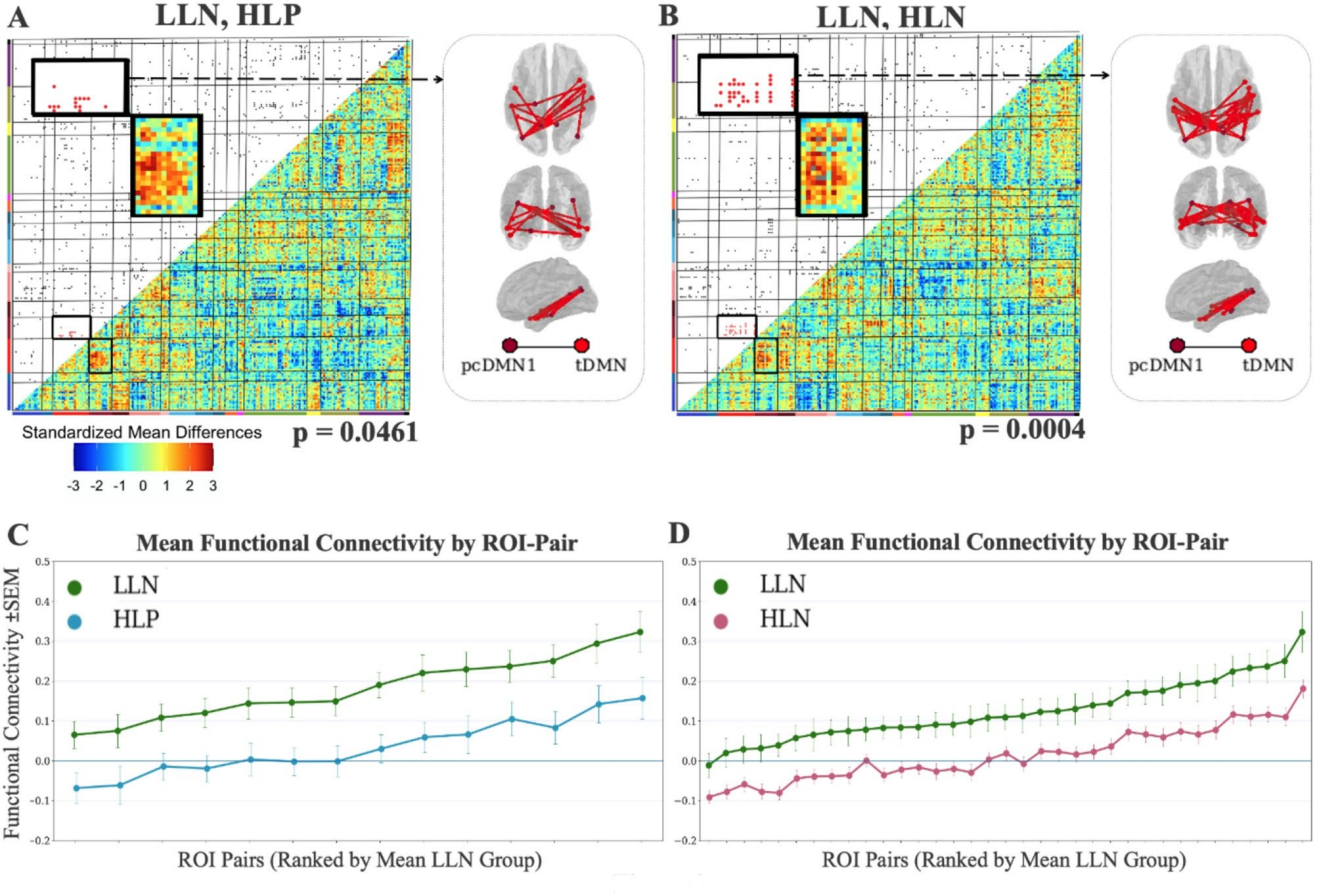
Enrichment analyses for pairwise Tukey-corrected t-tests characterize the pcDMN1-tDMN network pairs when comparing (**A**) the smaller LLN (*n* = 27) and HLP (*n* = 23) groups (*p* = 0.0461) and (**B**) the LLN and the larger HLN (*n* = 91) groups (*p* = 0.0004). Locations of implicated connections within the enriched network pair are visualized on dorsal, posterior, and lateral views of the brain. **C**,** D **The group mean functional connectivity profiles are remarkably consistent for pcDMN1 - tDMN ROI-pairs that show group differences: LLN > HLP (in **C**) and LLN > HLN (in **D**). The ROI-pairs are ordered by increasing LLN mean functional connectivity for clarity

Enrichment analyses (3-group, ANOVA F test-based, Fig. 5) identified the posterior cingulate default mode network 1–temporal default mode network pair (pcDMN1-tDMN, *p* = 0.007). No other network pairs had an enrichment p-value < 0.01. Functional connections in pcDMN-tDMN were used in our secondary vetting process [21] to predict ADOS CSS. The cross-validated mean squared error (MSE) for this test was significantly lower than expected by chance (*p* = 0.0396). fcMRI enrichment for post hoc Tukey-corrected t-test results (Fig. 6) on connections in the implicated pcDMN1– tDMN pair highlighted a difference between HLN and LLN (*p* = 0.0004), a marginal difference between HLP and LLN (*p* = 0.0461), and none between HLP and HLN (*p* = 0.2254). Difference matrices highlighting the pcDMN1-tDMN network pair for each comparison are displayed in Figure S2.

**Fig. 7:**
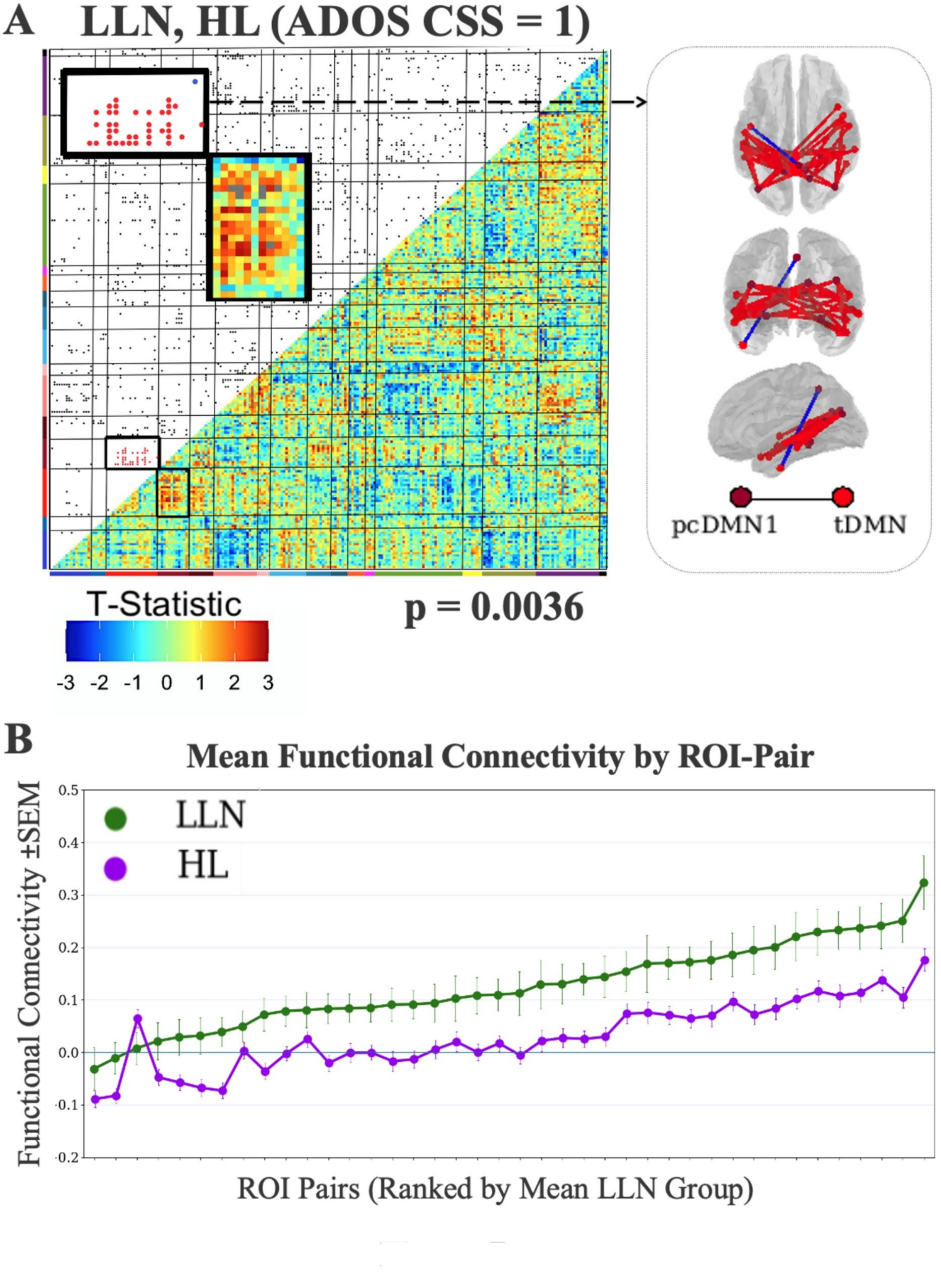
Enrichment analyses with student t-tests for the pcDMN1-tDMN network pair. **A **High-likelihood (HL) subjects compared with LLN (*p* = 0.0036). All subjects had a typical ADOS CSS = 1. Locations of implicated connections within the enriched network pair are visualized on dorsal, posterior, and lateral views of the brain. **B **Group mean functional connectivity profiles for pcDMN1 - tDMN ROI-pairs that show group differences: LLN > HL. The ROI-pairs are ordered by increasing LLN mean connectivity for clarity

In a more specific test of the familial hypothesis (i.e., that these fcMRI differences tracked familial likelihood status, not ASD symptom presence), we limited the analysis to subjects with ADOS CSSs = 1, indicating very few or no behavioral indicators of ASD (Fig. 7). t-test-based fcMRI enrichment (HL vs. LL) was significant for pcDMN1– tDMN connectivity (*p* = 0.0036). Further, the differences in mean connectivity between HL and LL subjects are remarkably consistent at the ROI pair level (Figs. 6 and 7): the average connectivity for LL subjects is higher than for HL subjects for all ROI pairs in pcDMN1– tDMN, and for all but one ROI-pair if the analysis is limited to subjects with ADOS CSS = 1.

Statistical testing for potential cross-group differences in network ROI compositions using Jaccard metrics produced no significant results.

## Discussion

Our findings suggest the novel hypothesis that familial liability for ASD manifests as reduced DMN connectivity at 24 months, while other processes operating on a more widely distributed set of brain functional connections correlate with the behavioral manifestation of ASD. This study tested for fcMRI correlates of ASD diagnosis and familial likelihood during a very narrow age window around the time when behavior consolidates to the extent that a diagnosis of ASD is possible. As such, the described effects associated with symptom emergence may be largely separate from later effects related to living with ASD. fcMRI enrichment, OODA, and SVM provided support for both familial (network-level enrichment results) and diagnostic (brain-wide OODA and SVM results) correlates.

fcMRI enrichment analysis identified a strong signal for the pcDMN1 – tDMN pair that was driven by lower functional connectivity strength for both HL groups versus that for LLN, with no HLP-HLN differences, consistent with a potential familial effect. We further explored that possibility by testing pcDMN1 – tDMN functional connectivity for HL versus LL subjects who had minimal ASD behavior as measured by the ADOS. This is a more rigorous test for familial effects since the comparison excludes HL subjects with significant ASD-related behavioral features. Differences in functional connectivity would then more likely be associated with familial status than overt, albeit subthreshold, ASD behavior. The results of these tests provide evidence for a familial effect, where the strength of functional connectivity between DMN networks is reduced in both ASD and non-ASD 24-month-olds at high familial likelihood for ASD. These DMN familial correlates could possibly represent a 24-month-old functional connectivity fingerprint of neural changes related to familial liability that are necessary but not sufficient for the development of ASD. Speculatively, some other biological or environmental process then leads to neural changes that are reflected as the distributed set of functional connections associated with diagnostic outcome in the HL subjects who do, versus do not, develop ASD.

Many studies in older subjects have reported functional connectivity findings in ASD associated with the default mode network (DMN) [1, 3, 22, 63]. The DMN is active during rest in typical subjects, reducing its activity during externally directed tasks, but task-based DMN deactivation has been reported to be absent in ASD (see Kennedy et al. [64]). As illustrated in Fig. 1 (see also [15]), the 24-month-old default mode networks appear disconnected relative to the unified default mode network in adults (cf [50, 65, 66]). Other accounts, however, support the existence of distinct DMN subnetworks in older subjects [66, 67]. We have reported DMN-related fcMRI correlates of dimensionally measured ASD behaviors in infants and toddlers: initiation of joint attention [15], walking and gross motor development [16], and restricted and repetitive behavior [17]. These and other findings allow us to articulate more specific hypotheses about the role that altered developmental patterning of DMN connectivity may play in the development of ASD. Recently [21], we hypothesized that atypically increased positive connectivity between control and default mode networks may be associated with increased ASD-related behaviors, such as poor motor functioning and increased restricted and repetitive behaviors. In addition, the timing for the emergence of these atypical connectivity patterns may possibly coincide with the development of DMN anti-correlation with other networks over the first years of life [8]. We will see how this hypothesis holds when tested with other analyses and in other datasets. Lombardo and colleagues [68] reported atypical DMN-visual connectivity in a subset of community-ascertained ASD toddlers who exhibited pronounced early social visual engagement difficulties, suggesting a potential link between DMN-visual connectivity and social visual engagement that warrants further study. Importantly, we have recently reported correlations between 6-month-old DMN to visual and somatomotor network connectivity and older ASD sibling social behavior [23], suggesting a link between these networks and familial liability for autism. Rolison and colleagues have also recently reported altered interhemispheric functional connectivity for the right posterior cingulate cortex, a DMN component, in infants at likelihood for ASD, that follows a familial pattern [69]. These familial likelihood associated findings in infants complement a result from Spencer and colleagues in older subjects that extends to unaffected siblings of autistic individuals [70] earlier reports [64] of reduced DMN deactivation in ASD during externally directed task performance.

In brain-wide analysis using OODA, we found an HLP-LLN difference that was not observed in statistical jackknife testing on correlation matrices. We reason this is the result of sample size limitations. We also found a nominally significant HLP-HLN difference that did not survive correction for multiple comparisons. HLN and LLN fcMRI matrices did not differ in this analysis. These brain-wide findings suggest a diagnostic outcome group-related pattern. We were not able to identify, in post hoc testing, specific networks driving these omnibus results. This suggests that more data may be needed to adjudicate between network-specific versus brain-wide-distributed diagnostic group fcMRI correlates, but the latter remain, for now, the face-level interpretation of the present results.

SVM was able to separate HLP and HLN toddlers with a high degree of accuracy using brain-wide fcMRI data. Importantly, this classifier was built *using only the HL sample*, and it correctly classified all *unused LLN matrices* as “ASD-negative.” This diagnosis-related pattern converges with our OODA results, and connections important for accurate SVM classification did not localize to specific networks. Finding highly accurate ASD diagnostic classification at 24 months is supportive of the predictive classification result we published in an overlapping sample [26]. The performance of the present classifier with data it did not train on has significant implications for our ongoing work to develop presymptomatic brain MRI-based ASD outcome classifiers that will generalize to unseen samples. We envision a future where a predictive classification result at six months of age [26] could triage infants into presymptomatic randomized controlled trials of yet-to-be developed interventions [29, 71].

We acknowledge several limitations, including that the sample is drawn from a pre-existing study for which data collection is now complete. Interval fcMRI processing advances allow these new cross-group tests. Brain-behavior correlation analyses with overlapping sets of subjects have been published previously [15–17] (hence, this paper’s focus on cross-group comparisons, which weren’t previously possible because of low Ns per group with motion-clean fcMRI data). We are collecting a new sample of 250 HL subjects. We are delayed because of the COVID pandemic, but we hope to be able to test for replication of the present results in several years. Sex ratio is imbalanced across groups in this study. Although male and female subjects were classified by SVM with similar accuracy, we will address this issue further in the larger, new sample that we are presently acquiring. The machine learning secondary vetting procedure for fcMRI enrichment is not completely free of potential issues with regard to classifier training contamination with test outcome information. Its intended use in this paper is to help ensure a reasonably robust characterization of connectivity differences that may be validated in independent samples, our own and others’. Our simulations suggest we will likely never have enough data for reliable individual-connection-level results in fcMRI enrichment (consistent with [72]). Our implementation of SVM asked questions at the brain-wide level, with the limitation of a low sample size relative to the number of features (> 26 K). We agree that the classifier is likely optimistic (e.g., one false positive). However, if this had been a spurious result driven by a class imbalance of number of cases to controls, or of sample size to number of features, we should have been able to develop similarly “good” classifiers in permuted data as well – and we did not. Furthermore, the crystallized classifier built with HL data also showed excellent performance using LLN data *not used in training*. As with individual fcMRI enrichment hits, the contribution of specific connections to accurate SVM cross-group classifications should be interpreted with caution. Functional connections contributing to accurate ASD diagnostic classification with SVM were widely distributed across the brain in a way that does not obviously converge with the connections implicated in our fcMRI enrichment analyses. It is not completely clear why fcMRI enrichment identified a familial but not diagnostic pattern that SVM identified, and OODA suggested. It is possible that our sample size was just on the edge of statistical power for each of these different methods. As such, they may have returned results reflecting their specific sensitivities: i.e., OODA and SVM for brain-wide tests, and fcMRI enrichment for network-level tests. These three methods might provide converging results with a larger sample.

In conclusion, we report a familial fcMRI difference between HL and LL subjects involving reduced DMN connectivity in 24-month-old infants with an older ASD-positive sibling. We also found evidence for ASD diagnostic differences involving functional connections that are widely distributed across many brain networks. Such results, if validated in independent samples, could serve as future biomarkers in quick-win/fast-fail treatment trials [30] where demonstrating intervention-related change in specific functional networks could facilitate the selection of candidate treatments for larger-scale clinical studies.

## Supplementary Information

Supplementary Material 1.

## Data Availability

Deidentified data have been submitted to the National Institute of Mental Health Data Archive (NDA).
