# Supplementary material for: Brain functional connectivity correlates of autism diagnosis and familial liability in 24-month-olds
Source: J Neurodev Disord. 2025 Jul 18;17:40. doi: 10.1186/s11689-025-09621-9 (PMC12275292; doi:10.1186/s11689-025-09621-9)
Supplement: Supplementary file 1 — Supplementary Material 1. [file 11689_2025_9621_MOESM1_ESM.docx]

**Supplemental**

**
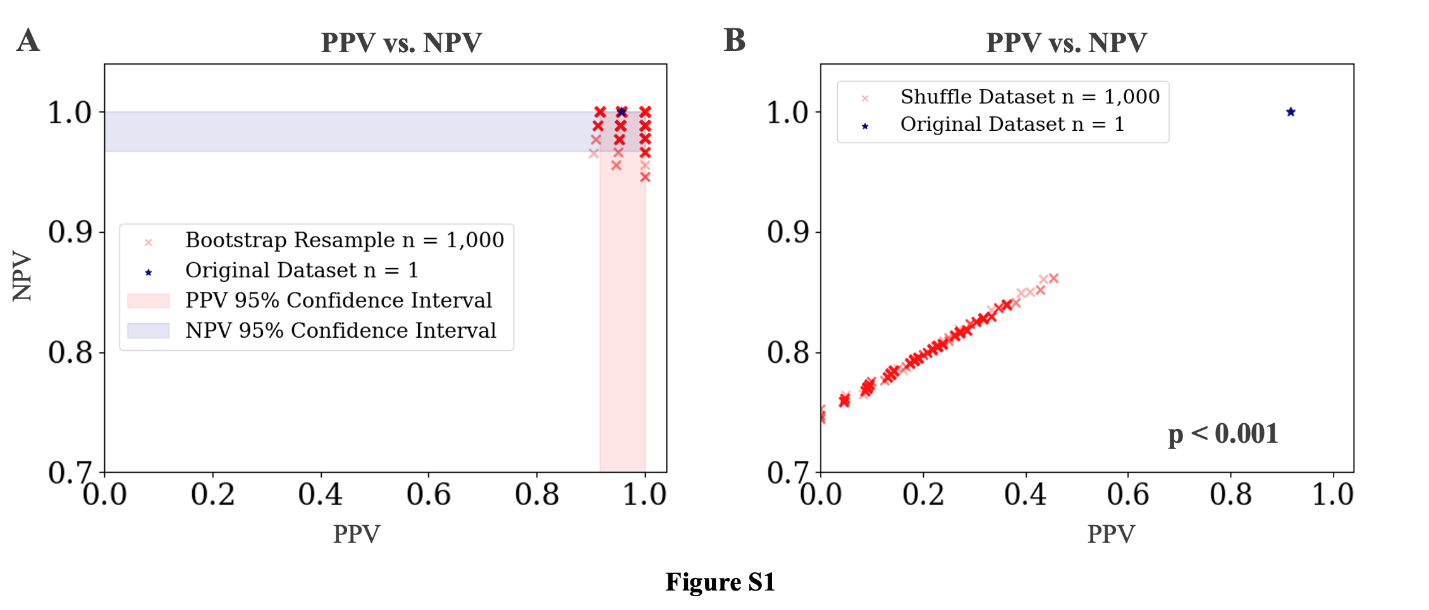
**

**Supplemental Figure 1.** Confidence intervals and randomization for SVM. **(A)** Positive predictive value (PPV) and negative predictive value (NPV) and 95% confidence intervals for the dataset and 1,000 bootstrapped resamples. **(B)** PPV and NPV for the actual dataset and randomly permuted data (1,000 permutations).


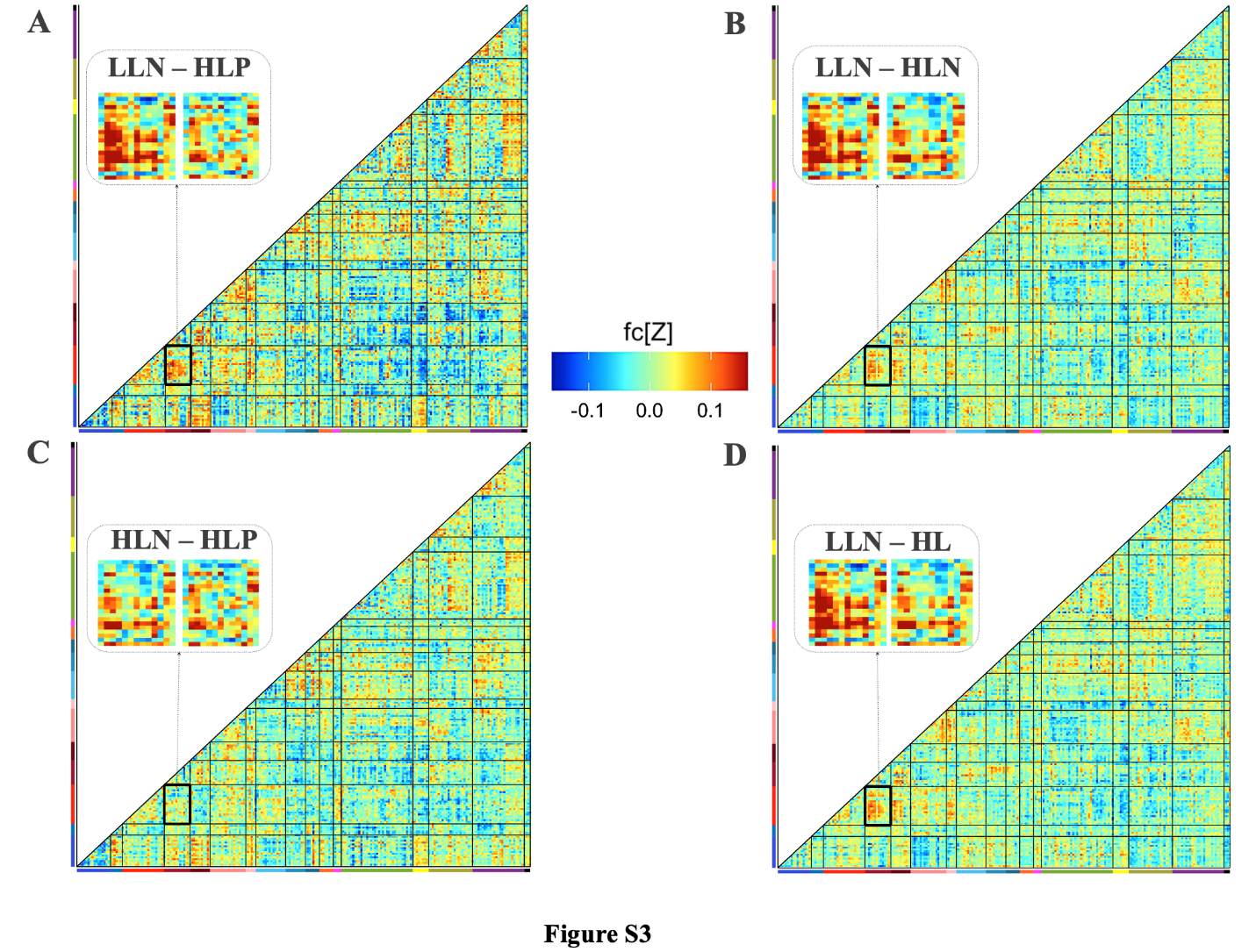


**Supplemental Figure 2.** Difference matrices were generated by subtracting the mean fcMRI adjacency matrix of one group from another, with pcDMN1-tDMN highlighted. The 230 ROIs are sorted by network (see figure 1). **(A)** HLP versus LLN. **(B)** HLN versus LLN. (C) HLP versus HLN. **(D)** Overall HL versus LLN.
